# Minimally invasive surgery for perihilar cholangiocarcinoma: a systematic review

**DOI:** 10.1007/s11701-019-00964-9

**Published:** 2019-05-02

**Authors:** L. C. Franken, M. J. van der Poel, A. E. J. Latenstein, M. J. Zwart, E. Roos, O. R. Busch, M. G. Besselink, T. M. van Gulik

**Affiliations:** Department of Surgery, Cancer Center Amsterdam, Amsterdam, UMC, University of Amsterdam, Amsterdam, The Netherlands

**Keywords:** Perihilar cholangiocarcinoma, Minimally invasive surgery, Systematic review

## Abstract

Minimally invasive surgery (MIS) is quickly becoming mainstream in hepato-pancreato-biliary surgery because of presumed advantages. Surgery for perihilar cholangiocarcinoma (PHC) is highly demanding which may hamper the feasibility and safety of MIS in this setting. This study aimed to systematically review the existing literature on MIS for PHC. A systematic literature review was performed according to the PRISMA statement. The PubMed and EMBASE databases were searched and all studies describing MIS in patients with PHC were included. Data extraction and risk of bias were assessed by two independent researchers. Overall, 21 studies reporting on a total of 142 MIS procedures for PHC were included. These included 82 laparoscopic, 59 robot-assisted and 1 hybrid procedure(s). Risk of bias was deemed substantial. Pooled conversion rate was 7/142 (4.9%), pooled morbidity 30/126 (23.8%), and pooled mortality rate 4/126 (3.2%). The only comparative study, comparing 10 robot-assisted procedures to 32 open procedures, reported a significant increased operative time and higher morbidity rate with MIS. The available evidence on MIS for PHC is limited and generally of poor quality. This systematic review shows that the implementation of MIS for patients with PHC is still in its infancy.

## Introduction

Perihilar cholangiocarcinoma (PHC) is an uncommon type of cancer with a bad prognosis. Surgical resection, usually entailing hilar resection with extended hepatectomy, is the only potentially curative treatment. These procedures are considered highly challenging due to the tumors’ proximity to the portal vein and hepatic artery [1]. Severe morbidity (Clavien–Dindo ≥ III) can rise up to 27.5–54% and mortality is high with rates of 1.4–18% [2–6]. The efficiency of surgical treatment of PHC has progressed in recent years with the surgical strategy changing from limited bile duct resections to resections including hepatectomy at the end of the twentieth century [3, 7]. This aggressive approach led to increased rates of *R*0 resections and 5-year survival [7, 8]. However, post-operative morbidity and mortality remain an issue.

Minimally invasive surgery (MIS) is increasingly being implemented in all types of hepato-pancreato-biliary resections including distal pancreatectomy and hepatectomy [9–11]. Promising results, inherent to a minimally invasive approach, such as faster functional recovery, less intra-operative blood loss, and less post-operative complications are frequently reported [10]. In liver surgery, laparoscopic and robot-assisted procedures have been increasingly used and show improved post-operative outcomes without compromising long-term oncological outcomes [11–13]. The extremely challenging nature of the procedure, the technical skills required, and the fear of oncological inefficiency have so far limited the adoption of MIS for PHC. Nevertheless, outcome of MIS for PHC has been reported [14]. A systematic review on MIS in patients with PHC is lacking.

### Objective

This systematic review aims to appraise the current literature on implementation and outcome of MIS for the treatment of PHC.

## Methods

The protocol of this study was registered in PROSPERO under number CRD42017074398. This systematic review is created in accordance with the Preferred Reporting Items for Systematic Review and Meta-Analyses (PRISMA) statement. We aimed to identify studies reporting on MIS in patients with PHC (i.e., Klatskin tumor). All study types in which a total laparoscopic (including hand assisted), robot-assisted and/or hybrid approach was described were eligible for inclusion. Studies without original data (e.g., reviews) and studies published in languages other than English were excluded. In case multiple eligible studies were published by the same group, the one with the highest number of cases was selected. To identify relevant studies, a search was conducted in PubMed and EMBASE on September 5th 2017. The search strategy was checked and approved by a clinical librarian. We used a combination of the following MeSH terms, keywords and search terms:

(“Laparoscopy”[Mesh] OR laparoscop* [tiab] OR “Hand-Assisted Laparoscopy”[Mesh] OR Hand Assisted Laparoscopy [tiab] OR “Robotic Surgical Procedures”[Mesh] OR robot* [tiab] OR “Minimally Invasive Surgical Procedures”[Mesh] OR Minimally Invasive OR hybrid [tiab]) AND (“Cholangiocarcinoma”[Mesh] OR cholangiocarcinoma* [tiab] OR Klatskin[tiab] OR “Bile Duct Neoplasms”[Mesh] OR Bile Duct cancer*[tiab] OR Bile Duct neoplasm*[tiab]).

### Data extraction and outcome measures

Two independent researchers (MJvdP and AL) screened abstracts and full texts for eligibility based on the inclusion and exclusion criteria. Any discrepancies were resolved by a third reviewer (MZ). Data were extracted using an extraction form and comprised the following variables: article details (author, title, demographics, year of publication, study type), amount of patients, preoperative characteristics (gender, age, type of Klatskin tumor according to the Bismuth–Corlette classification, symptoms, radiologic features), operative specifics (type of operation, technique, operative time, blood loss, conversion), and post-operative outcomes (morbidity, mortality, hospital stay, resection margins, hospital costs, recurrence and disease-free survival).

Two researchers (MJvdP and LCF) assessed the individual risk of bias on study level using the Newcastle–Ottawa Scale for Cohort studies and the Joanna Briggs Institute (JBI) Critical Appraisal Tools for Case Series and Case Reports. Discrepancies were resolved in a consensus meeting. Results from the risk of bias assessments for case series and case report are displayed in separate figures. Overall, risk of bias across studies is evaluated by assessing the selection bias, detection bias, attrition bias, and reporting bias.

## Results

### Study selection

**Fig. 1:**
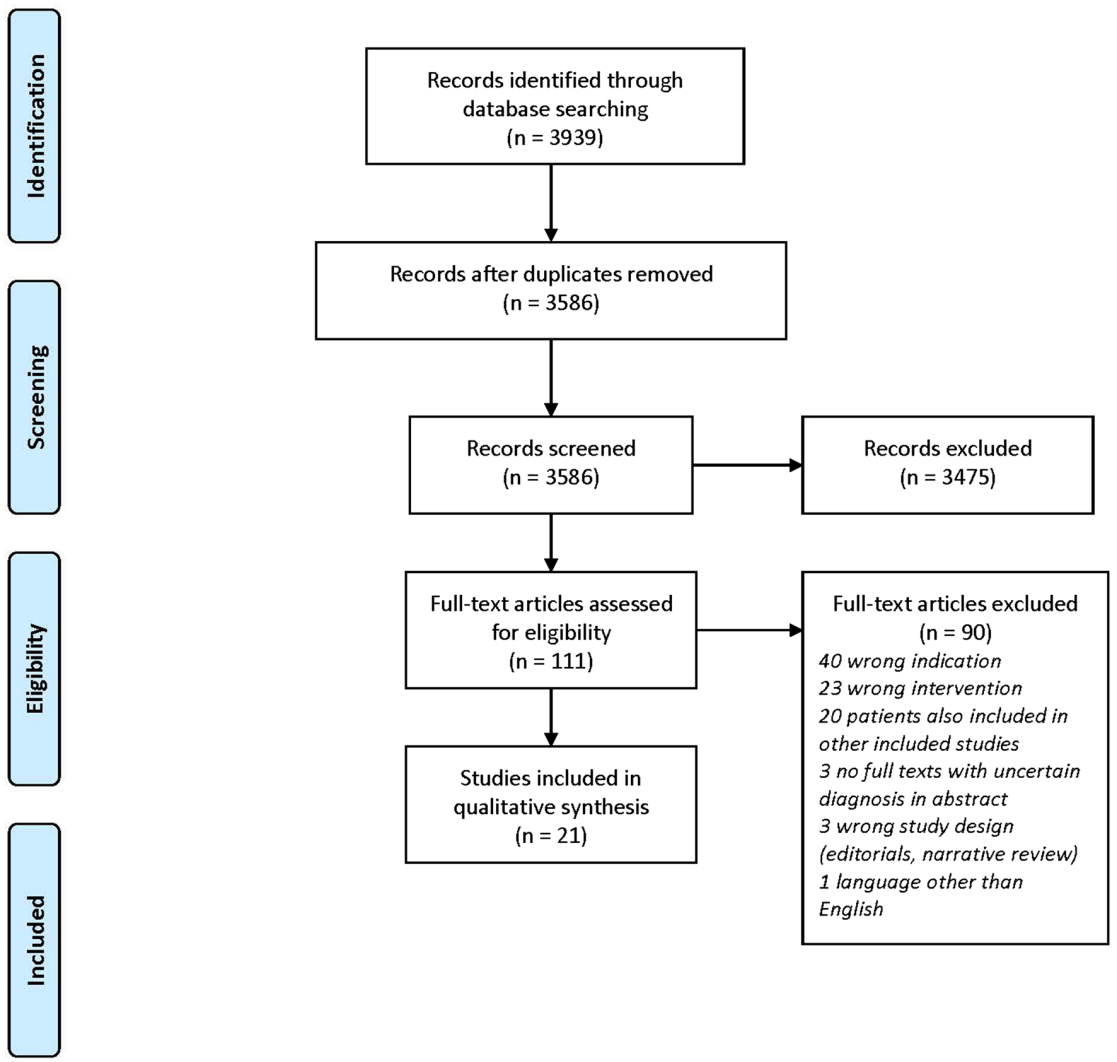
A flowchart of included studies

The initial search yielded 3939 studies. After removal of duplicates, a total of 3586 studies were screened for eligibility. This led to the screening of 111 full texts, which resulted in the inclusion of 21 studies [15–35]. Figure 1 displays the PRISMA flow diagram of study selection.

### Study characteristics

The 21 eligible studies included one retrospective comparative study, 6 case series, 5 case reports, 7 video abstracts, and 2 abstracts of posters. All studies had a retrospective design and the first study was published in 2010. All study characteristics of included studies are listed in Table 1. The only comparative study conducted by Xu et al., compared 10 robot-assisted procedures to 32 open procedures in patients with PHC. The largest series contributing to this systematic review consists of 44 patients [33]. As shown in Table 1, there were 14 studies (including 82 patients) that reported an accurate follow-up of more than 90 days with a maximum follow-up of 60 months [15–21, 23–25, 28, 31, 34, 35]. Six studies (11 patients) reported no follow-up after discharge [17, 26, 27, 29, 30, 32]. The follow-up period was unclear in one study (44 patients) [33].

**Table 1:** Study characteristics

First author	Year	Country	Study type	Approach	No. of pts.	Patient characteristics	Reported FU (months)
Xu et al. [15]	2016	China	Comparative study	Robotic	10 vs. 32	MIS: 8 men, 2 women, median 54 years, BC type II (1), IIIa (4), IIIb (1), IV(4)	Max 60
Chen et al. [16]	2013	China	Case series	Laparoscopic	36	27 men, 9 women, mean 66 years (45–85), BC type I (17), II (19)	4 pt LFU, 32 pt > 6
Yu et al. [17]	2011	China	Case series	Laparoscopic	14	8 men, 6 women, mean 55.7 years (51–57), imaging BC type I (8), II (6).	20 (7–33)
Li et al. [18]	2017	China	Case series	Laparoscopic	9	6 men, 3 women, median 62.7 years (50–74), BC type I (1), II (3), IIIb (2), IV (3), no vascular involvement	17 (6–42) 2 pt LFU
Lee et al. [19]	2015	Korea	Case series	Laparoscopic	5	5 men, median 63 years (43–76), BC type I (1), II (1), IIIa (1), IIIb (2)	8 (5–9)
Gumbs et al. [20]	2013	USA/Chile/France	Case series	Laparoscopic	5	Mean 73 years (66–79)	11 (3–18)
Quijano et al. [21]	2016	China	Case series	Robotic	1	–	3
Yu et al. [22]	2013	China	Case report	Laparoscopic	2	2 women, 54 + 60 years, BC type I	6–9 days
Puntambekar et al. [23]	2016	India	Case report	Laparoscopic	1	25-Year-old man, BC type II, no vascular involvement	6
Zhu et al. [24]	2014	China	Case report	Robotic	1	43-Year-old man, BC type IIIa	12
Machado et al. [35]	2012	Brazil	Case report	Laparoscopic	1	43-Year-old woman, BC type IIIb	18
Giulianotti et al. [25]	2010	USA	Case report	Robotic	1	66-Year-old man, PVE	8
Zhang et al. [26]	2018	China	Video abstract	Laparoscopic	1	BC type IIIa, no vascular involvement	11 days
Weaver et al. [27]	2010	USA	Video abstract	Laparoscopic	3	BC type IIIa	10–14 days
Efanov et al. [28]	2015	Russia	Video abstract	Robotic	1	65-Year-old man, BC type II, CHA replaced by and RHA adhered to tumor.	5
Nakahira et al. [29]	2015	Japan	Video abstract	Laparoscopic	3	–	19 days (16–23)
Chen et al. [30]	2017	Taiwan	Video abstract	Hybrid	1	BC type IV, 90-Year-old woman, no vascular involvement	9 days
Machado et al. [31]	2014	Brazil	Video abstract	Laparoscopic	1	58-Year-old woman, BC type IIIa	16
Ji et al. [32]	2011	China	Video abstract	Robotic	1	54-Year-old man	12 days
Zhou et al. [33]	2012	China	Abstract poster	Robotic	44	–	Unclear
Xu, J et al. [34]	2016	China	Abstract poster	Laparoscopic	1	68-Year-old man, BC type IIIa, no vascular involvement	14

*FU* follow-up, *LFU* lost to follow-up, *BC* Bismuth–Corlette

### Critical appraisal

**Fig. 2:**
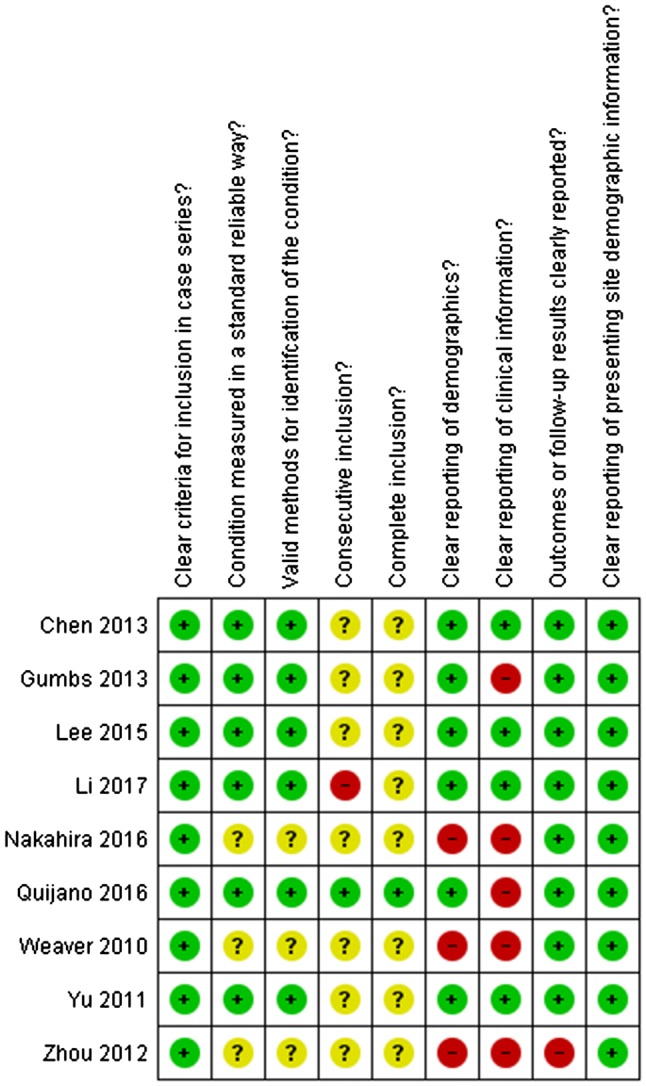
Risk of bias case series (JBI)

**Fig. 3:**
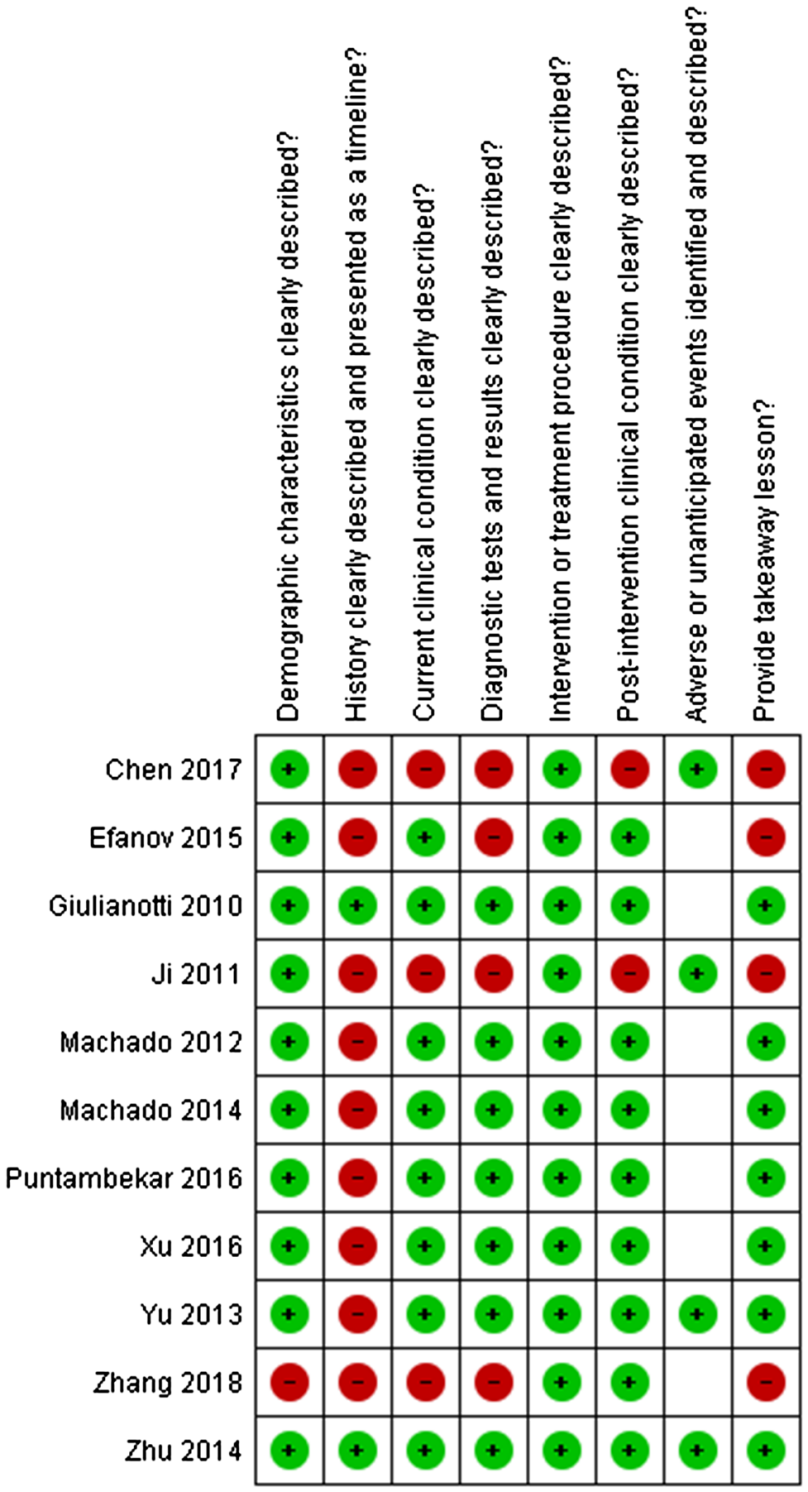
Risk of bias case reports (JBI)

The quality of the only comparative study [15] was assessed as poor on the Newcastle–Ottawa Scale, due to the lack of comparability and absence of controlling for confounders. Results of the Risk of Bias Assessment per study are displayed separately for case series and case reports in Figs. 2 and 3, respectively.

### Risk of bias

The majority of authors did not describe why they had subjected individual patients to minimally invasive procedures, causing a high risk of selection bias. None of the studies described that post-operative outcomes were assessed by an independent objective examiner. Also, a substantial proportion of the studies provided incomplete outcome data. These findings are highly suggestive for risk of detection and attrition bias. The inclusion of 11 case reports with no post-operative deaths and the lack of consecutive inclusion in case series suggest a publication bias.

### Patient and procedure characteristics

A total of 142 patients undergoing minimally invasive procedures for PHC were identified. Among 15 studies reporting on gender of their population, there were 59 men (69%) and 26 (31%) women. Reported age of included patients ranged between 25 and 90 years, with a mean age of 61 years. The most frequently reported presenting symptom was jaundice. Thirteen studies described Bismuth–Corlette stage (BC) of their study population, including 29, 32, 12, 6, and 8 patients with type I, type II, type IIIa, type IIIb, and type IV tumors, respectively. Detailed patient demographics per study are listed in Table 1.

The 142 included procedures contained 82 laparoscopic, 59 robot-assisted, and 1 hybrid procedure(s). The first minimally invasive procedure for PHC was described by Chen et al. [16], performed in 2000. The da Vinci^®^ Robotic Surgical System was used for the majority of robot-assisted procedures. External bile duct resection only was performed in 63 cases. Additionally, external bile duct resection was combined with a major hepatectomy in 35 patients (15 left hemihepatectomies, 8 right hemihepatectomies, 10 extended right hemihepatectomies, and 2 extended left hemihepatectomies). In the remaining 44 patients, the external bile duct resection was combined with caudate lobe resection or minor hepatectomy.

### Operative outcomes

Due to high heterogeneity across studies and major differences in population and procedures, the operative time, hospital stay, and blood loss varied widely. Generally, operative time of robotic procedures was longer compared to laparoscopic procedures. Across all included procedures average operation time was 381 min (range 205–1010 min) and average blood loss was 398 ml (range 43–2169 ml). Overall, the conversion rate to open surgery was 4.9% (7/142). The shortest reported hospital stay was 3 days, while the longest post-operative admission was reported to be 58 days. The average hospital stay across all studies was 10.8 days. Xu et al. [15] reported that the robotic procedures showed a longer operative time, hospital stay and more blood loss compared to open surgery (703 vs 475 min, 16 vs. 14 days, 1360 vs 1014 ml, respectively). Differences in hospital costs were only described by Xu et al, showing significantly higher costs for the robotic approach compared to the open approach (27,427 ± 21,316 versus 15,282 ± 5957 dollar, respectively).

The pooled post-operative morbidity rate was 30/126 (23.8%) (see Table 2). Although the follow-up duration was unclear in one included study conducted by Zhou et al. [33], their reported morbidity of 8/44 (18.2%) and mortality of 1/44 (2.7%) were included in the pooled morbidity and mortality because data on post-operative outcomes were scarce. The most frequently reported complication was bile leakage, overall 15 times described. Additionally, one post-hepatectomy liver failure, four peritoneal/pleural effusions, two thromboses (portal vein and lower extremities), one hemorrhage, and one intra-abdominal fluid collection were described. Overall 90-day mortality was 3.2% (4/126), calculated with data from 13 studies with mortality ranging between 0 and 22%. The only comparative study showed a significant difference in morbidity between the open and robotic approach in favor of the open approach: 9/10 (90%) patients undergoing robotic surgery experienced complications compared to 16/32 (50%) in the group undergoing open surgery. Mortality did not differ significantly between open (6.3%) and robotic surgery (10%) [15]. Morbidity and mortality per study are listed in Table 2. Resection margins were reported in 57 cases, of which 46 R0-resection (79.3%), seven R1-resection, and two R2-resections.

**Table 2:** Operative characteristics and outcomes

Author, year	No. of procedures	Type of resection	Operation time (min)	Blood loss (ml)	Hospital stay (days)	Conversion	Pathology	Morbidity	Mortality
Xu, Y [15]	10 vs. 32	Robotic-assisted LHH (4), RHH (4), ERHH (1) + EBDx, LNx, RYHJ vs. open traditional approach	703 ± 62 vs. 475 ± 121	1360 ± 809 vs. 1014 ± 811	16 (9–58) vs. 14 (4–54)	0/10	3 R1, 5 R0 vs unknown	9/10 (90%) (=/> CD gr III 3/10, bile leakage 4, pleural/peritoneal effusion 2, PHLF 1, PV thrombosis 1, hemorrhage 1, DVT 1) vs. 5/32 (16%) (=/ > CD gr III 2/32)	90 days 10% vs 6%
Zhou [33]	44	23 tumor resection + robotic LHH (3), GD-bridged biliary reconstruction (3), RYHJ (16), biliary reconstruction (1) 21 palliative biliary external drainage (9 external biliary drainage, 12 T-tube biliary drainage)	–	–	–	–	–	8/44 (18%)	2%
Chen [16]	36	EBD*x*, CL*x*, total laparoscopic RYCJ (end-to-side CJS)	205.3 ± 23.9	101.1 ± 13.6	5.9 ± 2.1	0/36	–	Bile leakage 1/36 (3%)	0%
Yu [17]	14	7 lap EBD*x*, LN*x*, RYCJ 5 Lap part hepatectomy (segm I, IV or V), HJ 1 lap EBD*x* + external biliary drainage 1 combined partial liver resection + HJ	305 (200–1000)	386 (200–1000)	BC I: 9 (6–22), BC II: 19 (9–25)	0/14	7 R0 3 R0, 2 R1 R2 R2	Bile leakage 5/14 (36%)	90 days 0%
Li [18]	9	Lap 2 CLx, 2 LHH, RYHJ (2 laparoscopic, 4 under direct visual observation, 3 hand-assisted)	438 (330–540)	503 (150–850)	15.7 (10–27)	3/9 (33%)	R0 9/9	Biliary fistula 2, peritoneal effusion 2 (all conservative)	30 days 11%, 90 days 22%
Lee [19]	5	Total laparoscopic hilar resection + bilioenteric anastomosis (1), + laparoscopic-assisted HJ, 3 laparoscopic EHH left (2), right (1).	610 (410–665)	650 (450–1300)	12 (9–21)	0/5	1 R1, 4 R0	Bile leakage 1/5 (20%) (percutaneous drain)	90 days 0%
Gumbs [20]	5	Lap EBD*x* (3) + RHH (1), LHH (1), RYHJ or RYCJ	–	240 (0–400)	15 (11–21)	1/5 (20%)	1 R1, 4 R0	No bile leakage	90 days 0%
Quijano [21]	1	Robotic LHH, hilar LN*x*, right side biliary resection, HJ	510	1000	16	1/1	R0	60 days: intra-abdominal fluid collection(CD gr II)	60 days 0%
Yu [22]	2	Single-incision lap segmental BD*x*, LN*x*, RYCJ, entero-enteric anastomosis	300, 350	350, 400	6, 9	0/2	R0	Bile leakage 1/2 (50%)	No FU after discharge
Puntambekar [23]	1	Lap EBDx, RYHJ	240	150	6	0/1	R0	None	90 days 0%
Zhu [24]	1	Staged procedure 1) Robotic drainage, dissection of right hepatic vessels, right hepatic vascular control device 2) RHH	–	700	14	0/1	R0	None	DFS 12 months
Machado [35]	1	Lap EBD*x,* LHH, LN*x*, video-assisted bilioenteric reconstruction	300	–	7	0/1	R0	None	DFS 18 months
Giulianotti [25]	1	Robotic ERHH with left RYHJ	540	800	11	1/1	R0	None	DFS 8 months
Zhang [26]	1	Pure lap ERHH, LN*x* and left HJ	590	300	11	0/1	R0, 2 cm	None	No FU after discharge
Weaver [27]	3	Lap ERHH (3), LN*x*, RYHJ	–	–	3 or 4	0/3	R0	–	No FU after discharge
Efanov [28]	1	Robot-assisted LHH, EBD*x*, LN*x*, HJ	960	300	30	1/1	R0	Bile leakage (conservative)	DFS 5 months
Nakahira [29]	3	Lap LN*x*, ERHH, end-to-side endoscopic HJ	867 (range 853–1010)	100 (43–400)	19 (16–23)	0/3	–	–	‘Post-operative’ 0%, no FU
Chen [30]	1	Lap LHH, regional LN*x* and laparoscopic-robotic RYHJ	465	150	9	0	cis, R0	None	No FU after discharge
Machado [31]	1	Totally lap RHH, LN*x*, RYHJ	400	400	10	0/1	R0	None	DFS 16 months
Ji [32]	1	Robotic-assisted laparoscopic LHH, RYHJ	600	600	12	0/1	R0	Bile leakage (conservative)	No FU after discharge
Xu, J [34]	1	Laparoscopic RHH, hilar LN*x*, RYHJ	420	400	8	0/1	R0	None	DFS 14 months

*LHH* left hemihepatectomy, *RHH* right hemihepatectomy, *ERHH* extended right hemihepatectomy, *ELHH* extended left hemihepatectomy, *HJ* hepaticojejunostomy, *CJ* choledochojejunostomy, *RY* Roux-en-Y, *CL* caudate lobe, *(E)BD* (external) bile duct, *x* resection, *LNx* lymphadenectomy, *lap* laparoscopic, *PHLF* post-hepatectomy liver failure, *PV* portal vein, *CD gr* Clavien–Dindo grade, *FU* follow-up, *LFU* lost to follow-up, *DFS* disease-free survival, *DVT* deep venous thrombosis of lower extremities, *CHA* common hepatic artery, *RHA* right hepatic artery

## Discussion

In this first systematic review on MIS in patients with PHC, we found that this field is still in its infancy. A total of 142 laparoscopic and robot-assisted procedures in patients with PHC were reported. Case series and case reports included in this study show that laparoscopic and robotic external bile duct resection combined with (hemi)-hepatectomy is technically feasible in highly selected patients with PHC in experienced hands. However, results from the only comparative study that was identified appear to be in favor of the open approach.

The only comparative study, by Xu et al, included in this systematic review showed that MIS is inferior to the open approach in patients with PHC in terms of operative time, blood loss, morbidity and mortality [15]. Clearly, a learning curve effect cannot be excluded. All other included studies were non-comparative and small, retrospective case series or case reports. This introduces a high risk of selection and publication bias. For example, combining results from all included case reports and case series showed a conversion rate of 5% (7/142). Nevertheless, in laparoscopic major liver resection, literature shows a range of conversion rate between 9 and 42% [36] and even in laparoscopic cholecystectomy the conversion rate remains between 5 and 10% [37]. The conversion rate of 4.9% seems thus extremely low. Furthermore, the total of 4 deaths and 30 complications among 126 patients suggests an overall 90-day mortality of 3% and a post-operative morbidity rate of 24%. Mortality and morbidity of open surgery in patients with PCH are infamously high and reported up to 18 and 68%, respectively [2, 4]. Looking at duration of hospitalization, the average hospital stay for patients undergoing open surgery for PHC varies between 16 [38] and 23 days [39]. Comparing this with the average hospital stay for MIS in this review of 10.8 days, it may appear that MIS results in a shorter hospital stay. These comparisons with literature suggest a benefit of MIS compared to open surgery, but should be interpreted with extreme caution: these preliminary results may not be truly representative of current practice and are very likely to be influenced by strict patient selection and may represent only the favorable outcomes. Furthermore, all included studies derived from high-volume HPB units with surgeons experienced in minimally invasive HPB surgery. Therefore, results cannot be widely reproduced and should limit the use of MIS for this specific patient population to only those experienced centers.

R0 resection was achieved in almost 80% of patients. A large series consisting of 331 open resections of PHC shows that only in 59% of the cases R0 resection could be achieved [40]. This most likely confirms the presence of selection bias. On the other hand, the previously described meta-analysis on laparoscopic hepatectomies showed no significant differences in resection margins either [10]. Due to a lack of long-term follow-up, the effect of MIS on oncological outcomes remains uncertain.

One of the major limitations of this study was the above-described substantial risk of bias. Because of this significant risk of selection and publication bias, results presented in this review based on these case series and case reports have a potential bias towards a good result. Also, all studies included in this systematic review were retrospective, small and generally of poor quality. Another limitation was the high heterogeneity among patient cohort and procedures.

This systematic review identified preliminary results from low-quality studies from highly experienced centers on MIS in PHC. It remains to be seen if the inherent benefits of MIS are applicable in this highly complex patient population and further research should focus on a safe implementation. To secure a safe and transparent implementation of MIS in PHC, patients should only be treated within prospective studies in highly selected centers.
